# Economic Impact of Tissue Testing and Treatments of Metastatic NSCLC in the Era of Personalized Medicine

**DOI:** 10.3389/fonc.2014.00258

**Published:** 2014-09-22

**Authors:** Donna M. Graham, Natasha B. Leighl

**Affiliations:** ^1^Department of Medicine, Division of Hematology and Oncology, Princess Margaret Cancer Centre, University of Toronto, Toronto, ON, Canada

**Keywords:** metastatic NSCLC, economic impact, tissue testing, personalized medicine, medical economics

## Abstract

A paradigm-shift in the management of non-small cell lung cancer (NSCLC) has resulted in many new therapies becoming available for patients with advanced disease. Stratification of treatment by histologic and molecular subtype is recommended to obtain the greatest clinical benefit for patients while minimizing adverse effects of treatment. However, these advances in diagnosis and treatment of NSCLC have come at a financial cost. This review highlights the economic impact of screening for molecular abnormalities and targeted treatment for advanced NSCLC. Major determinants of cost are drug acquisition and molecular testing. As technologies advance, molecular testing costs may reduce. However, we must collaborate with payers and manufacturers to ensure that high drug costs do not limit patient accessibility to potentially beneficial treatment.

## Introduction

Increasing understanding of the biology of cancer has resulted in strategies to personalize therapy for patients. In advanced non-small cell lung cancer (NSCLC), these advances have led to stratification of treatment by histological and molecular subtype to obtain the greatest clinical benefit, while minimizing adverse effects of treatment (1–3). However, these innovations in diagnosis and treatment of NSCLC have come at a financial cost. Where cure is not an option, the impact of cost is a significant consideration in provision of cancer care.

## Treatment for NSCLC

The SWOG 9509 (4) and ECOG 1594 (5) studies established platinum-based doublet chemotherapy as the treatment of choice in advanced NSCLC. These studies did not demonstrate benefit between treatment regimens for any subgroup analyzed. However, comparison of pemetrexed with docetaxel as second-line therapy (6), and a subsequent randomized trial comparing the combination of pemetrexed/cisplatin with gemcitabine/cisplatin as first-line treatment (7), highlighted a clinical benefit for pemetrexed in patients with non-squamous histology, giving the first suggestion that NSCLC can no longer be treated as one disease. Further attempts to improve outcomes included the addition of bevacizumab to platinum-doublet chemotherapy. This combination resulted in hemoptysis when used to treat patients with squamous cell carcinoma (8), resulting in selective treatment of patients with non-squamous histology only. A modest survival benefit of two months was seen in the bevacizumab arm with overall survival of 12.3 months compared with 10.3 months for the chemotherapy alone arm (9) and 4 months in the adenocarcinoma subgroup.

Further therapeutic options became available with the emergence of epidermal growth factor receptor (*EGFR*) tyrosine kinase inhibitors (TKIs). The presence of activating mutations in exons 18, 19, 20, and 21 of *EGFR* in NSCLC [in 15% of adenocarcinoma (10)] predicts for improvements in progression-free survival, response, and quality of life with the use of EGFR TKIs for this subpopulation of patients compared to traditional chemotherapy (10–12). In addition, the presence of the echinoderm microtubule-associated protein-like 4-anaplastic lymphoma kinase (*EML4-ALK*) fusion gene in 2–7% of cases of NSCLC (13) is a target for therapy with crizotinib with enhanced response rates and progression-free survival when compared to second-line chemotherapy in pre-treated patients, and more recently first-line treatment (3). These targeted therapies have dramatically changed the diagnosis and treatment of NSCLC.

## Costs of Lung Cancer Management

Increasing costs of cancer management are a global issue. The estimated cost of cancer care in the United States (US) was $124.57 billion in 2010, with a minimum estimated cost of lung cancer care being $12.12 billion. Using the most conservative estimates, this cost was predicted to increase by 25% to $15.19 billion by 2020 (14). However, this does not account for changes in treatment strategy and the introduction of novel agents. Canadian data have shown that the proportion of patients receiving systemic therapy has doubled from 18.1 to 37.5% from 1997 to 2007 but that the treatment costs tripled during this interval (15). Cost is of major concern to patients and payors, with medical debt being the most common cause of personal bankruptcy in the US (16). Molecularly targeted agents, while providing clinical benefit, carry a high price tag. The monthly cost of the EGFR TKI erlotinib is $2,847CAD (Canadian dollars), and the ALK TKI crizotinib costs $10,400CAD for a month’s supply (17). Additional expenses may also apply, including overhead costs, within certain countries.

With the expanding use of targeted therapies in this population, even greater increases in the cost of lung cancer treatment are anticipated. In addition, the cost of further diagnostic testing to aid treatment selection will escalate costs in the management of lung cancer. Obtaining value for money when prescribing expensive medications is critical for patients, payors, and society.

### Diagnostic testing

As histology and molecular subtype are such critical determinants of cancer treatment, adequate tissue sampling is vital. Approximately 70% of NSCLC is diagnosed at an advanced stage, usually by small biopsy sampling rather than surgical resection. International guidelines have recommended routine immunohistochemical staining (IHC) of all NSCLC for diagnosis, histologic subtype and molecular testing for *EGFR* mutation, and *EML4-ALK* fusion for patients with advanced NSCLC (18–20). With small tissue or cytology samples, the diagnostic yield may be compromised, resulting in a requirement for re-biopsy to obtain more tissue to accurately provide a diagnosis. In the IPASS study, 44% of patients did not have available tissue for molecular testing (12), similar to 55% of patients in the BR.21 study (2). In addition, the tumor content may be insufficient for molecular testing (21). Amount of tissue required and labor intensiveness depend on the techniques employed, e.g., IHC requires less tissue and is less costly than fluorescent *in situ* hybridization (FISH) or sequencing, costing $40CAD compared with $388CAD for FISH (22). Therefore, the availability of tissue and method of testing are of clinical and economic importance.

Standardized IHC is recommended for diagnosis of NSCLC and the determination of histologic subtype. The current gold standard for *EML4-ALK* testing, used in initial clinical studies as a companion diagnostic tool, is the use of a break-apart FISH assay (Vysis ALK Break Apart FISH Probe, Abbott Molecular Inc., Des Plaines, IL, USA). However, reverse-transcriptase polymerase chain reaction, IHC, chromogenic *in situ* hybridization (CISH), and other techniques may also be used. The most reliable of these alternative methods is IHC, due to improved sensitivity and specificity of the antibodies (23). IHC has been shown to correlate with FISH in several studies (24, 25), providing a far less costly and more easily accessible method for preliminary detection of *EML4-ALK* fusion, which may subsequently be confirmed with FISH (26). *EGFR* mutation testing can be performed using Sanger sequencing, and other less labor-intensive methods of *EGFR* mutation testing have been developed, which may have even greater sensitivity (27, 28). Multiplex assays and next-generation sequencing in lung cancer samples are tested for several genomic aberrations simultaneously and usually include *EGFR* genotyping.

Personalized therapy relies on the presence of a predefined clinical, pathological, or molecular biomarker. Biomarkers can be incorporated into drug development by different methods. Where a biomarker is integral to the drug development process, the population are screened and pre-selected for treatment on the presence of this biomarker. In order for this to be a valid strategy, robust preclinical data must strongly support this methodology. Crizotinib (an ALK, ROS-1, and MET inhibitor) is an example of a drug that was developed using an integral approach (29). An *a priori* hypothesis of efficacy in patients with *EML4-ALK*+, *ROS-1*+, and *MET* amplified tumors was used to enroll only these subpopulations to the study. This trial design led to accelerated approval for this agent, where the relatively low frequency of *EML4-ALK* in the population of NSCLC may have otherwise resulted in a negative outcome. However, there is a concern that this approach may miss activity in biomarker-negative patients who may potentially benefit from an agent, if they are excluded from clinical trials. In addition, the cost of identifying the target population in this type of study is not accounted for.

Alternatively, a biomarker may be integrated into trial design, allowing both biomarker-positive and -negative patients to receive treatment, thereby enabling assessment of benefit in both groups. In this case, all patients are tested for the presence of the biomarker, and analysis of the subpopulation of interest occurs retrospectively. This was the case with the EGFR TKIs, where the biomarker of interest was initially thought to be EGFR protein expression (30) but pre-specified subgroup analysis confirmed a greater benefit for this therapy in patients with the presence of *EGFR* mutation in the tumor (12, 31–33).

## Economic Analyses

Economic analyses aim to contextualize the cost of healthcare services by providing a measure of the cost and consequences for different treatments. The gold standard for oncology is the cost–utility analysis. Results are commonly presented as incremental cost-effectiveness ratios (ICER) and cost per quality-adjusted life year (QALY) to give a measure of the value of the intervention based on clinical benefit and costs (34). The quality of an economic assessment is often driven by the existing clinical data to support the intervention (35).

Different paths of drug development become important in economic analyses when considering the methods by which the intervention in the target population and the comparator is defined. There are different approaches to evaluate the cost of personalized medicine. It is possible to focus only on the target population and compare the intervention with other comparators in that group. However, the cost of identifying the target population through molecular testing will not be incorporated in this design, thereby potentially underestimating total cost of therapy. An alternative approach would be to compare a strategy of testing for the target biomarker in the entire population followed by treatment of the target population, with a strategy involving no biomarker testing and standard of care therapy. However, this relies on availability of an accurate assay for biomarker assessment in order to identify the true target population as a proportion of the population as a whole. An effect on small target populations may have minimal change in outcome for the population as a whole, especially if the target population is very small, e.g., 1–2%. Also, improvements in technology may result in a change of testing strategy and modified costs in the future. Another approach may be to separate the test and treat component of the analysis (26).

### EGFR TKIs

These agents were developed before the optimal target population was defined. Thus, early trials in lung cancer involved unselected advanced NSCLC patients.

An economic analysis of erlotinib in previously treated otherwise unselected patients with NSCLC was performed by the NCIC clinical trials group (NCIC CTG) based on data from the NCIC CTG BR.21 study. An ICER of $94,638CAD per life-year gained (LYG) (95% confidence interval of $52,359–429,148CAD) was identified (36). Exploratory analysis identified that treating never-smokers and patients with high tumoral *EGFR* gene copy number were the most cost-effective strategies. Interestingly, in patients with sensitizing *EGFR* mutations, treatment was associated with an ICER of $138,168CAD/LYG compared with $87,994CAD/LYG for patients with *EGFR* wild-type tumors. This likely reflects the small survival benefit noted in this study for both groups and the shorter duration of therapy in patients with *EGFR* wild-type tumors.

Over time, we have learned that patients with *EGFR*-mutated advanced NSCLC derive the greatest benefit from EGFR TKI therapy, which is superior to chemotherapy in terms of response rate, quality of life, and progression-free survival, although not in overall survival due to crossover in clinical trials, with a recent exception (35). Given this clinical benefit, a number of analyses have been performed to assess cost-effectiveness in this setting (Table 1). Using platinum-doublet chemotherapy as a comparator, a CE estimate of £59,216–70,390/QALY was calculated for first-line gefitinib in a British study (37), but was not considered cost effective at standard willingness-to-pay thresholds. A number of studies have also investigated the cost-effectiveness of EGFR TKI treatment with *EGFR* mutation testing included. Based on the IPASS study (12), a Singaporean study suggested that first-line treatment of *EGFR*-mutated NSCLC with gefitinib was a cost-effective strategy with a CE estimate of $77,160 Singaporean dollars/QALY (38) compared with carboplatin/paclitaxel, carboplatin/pemetrexed, or carboplatin/pemetrexed/bevacizumab. Of note, their model included second-line gefitinib for patients treated with initial chemotherapy irrespective of *EGFR* genotype.

**Table 1:** **Cost-effectiveness studies of first-line EGFR TKI therapy**.

Author	Type of study	EGFR TKI and comparator	Model	Cost of testing	Perspective	ICER per QALY	Cost- effective?	Remarks
Brown et al. (37)	Cost-effectiveness analysis	Gefitinib compared with platinum-doublet chemotherapy	Decision model comparing gefitinib with carbo/tax in patients with *EGFR* mutation positive disease	No	National Health Service	£59,216–70,390	No	Clinical data from IPASS: (12)
de Lima Lopes et al. (38)	Cost–utility analysis	Gefitinib compared with carbo/gem Subset analysis of gefitinib as second-line Assumed 60% with *EGFR* mutation	Decision tree with testing versus no testing and multiple lines of treatment. Test positive: gefitinib, carbo/gem, BSC Test negative: Carbo/gem, BSC No testing: Carbo/gem, gefitinib, BSC	Included $380	Singaporean health care system, 2010 Singapore dollars	$77,160	Yes	Clinical data from 3 trials: IPASS: (12);WJTOG 345: (32); C000000376: (33)
Handorf et al. (39)	Cost-effectiveness analysis	Erlotinib compared with carbo/tax, carbo/pem, and carbo/pem/bev	Decision analytic model with testing versus no testing and re-biopsy included Test positive: erlotinib Test negative: platinum-based chemotherapy No testing or insufficient tissue on repeat biopsy: platinum-based chemotherapy	Yes	Payer’s perspective	$110,658 for carbo/tax test and treat $122,234 for carbo/tax re-biopsy $180,665 for carbo/pem $359,619 for carbo/pem/bev	Yes	Re-biopsy strategy included: assumed 15% yielded insufficient tissue
Brown et al. (40)	Cost-effectiveness analysis	Gefitinib compared with platinum-doublet chemotherapy	Decision model comparing gefitinib with cis/tax, carbo/tax or cis/doc	No	UK National Health Service and Personal Social Services	Mean £35,700 (range £59,216– 70,390)	No	Clinical data from IPASS: (12, 31); WJTOG 345: (32); C000000376: (33); Mean negotiated NHS costs included
Wang et al. (41)	Cost-effectiveness analysis	Erlotinib compared with carbo/gem	Markov model comparing carbo/gem for 4 cycles with erlotinib until progression	No	Chinese health care system, 2010 US dollars	$85927.41 (range $58,584.57–336,404.20)	Yes	Clinical data from OPTIMAL trial: (42) Cost of erlotinib included only for first 7 cycles (from cycle 8 or month 5 onward cost is zero due to donations from Roche China)

*EGFR TKI, epidermal growth factor tyrosine kinase inhibitor; ICER, incremental cost-effectiveness ratio; LY, life years; QALY, quality of life year; carbo/gem, carboplatin and gemcitabine chemotherapy; BSC, best supportive care; carbo/tax, carboplatin and paclitaxel; carbo/pem, carboplatin and pemetrexed; carbo/pem/bev, carboplatin, pemetrexed and bevacizumab; cis/tax, cisplatin and paclitaxel; cis/doc, cisplatin and docetaxel*.

The potential for insufficient diagnostic tissue available for *EGFR* mutation testing (2, 12) prompted a study in which either no lung adenocarcinoma patient samples were tested (all received first-line chemotherapy), a second testing scenario where half of the patients had sufficient tissue for *EGFR* testing, or a third scenario where half of the patients had repeat tumor biopsy for *EGFR* testing (although 15% still had insufficient tissue after re-biopsy). First-line erlotinib therapy resulted in an ICER of $110,658/QALY gain compared with carboplatin/paclitaxel with the testing strategy and $122,234/QALY using the re-biopsy strategy. With carboplatin/pemetrexed as a comparator, the ICER for the repeat biopsy strategy was $180,665/QALY; adding bevacizumab increased the ICER significantly to $359,619/QALY, in excess of commonly accepted thresholds for cost-effectiveness (39). A recent study from the perspective of the Chinese healthcare system investigated the cost-effectiveness of first-line erlotinib compared with platinum-doublet chemotherapy in advanced *EGFR* mutation positive lung cancer patients based on outcomes from the OPTIMAL trial (42). Treatment with upfront erlotinib was deemed cost effective with an ICER of $85,927.41USD/QALY gained. Of note, this analysis assumed that after the first 5 months (seven cycles) of therapy, subsequent erlotinib would be donated by Roche China.

A U.S. study demonstrated a modest budget impact of *EGFR* mutation testing and erlotinib as first-line therapy for patients with *EGFR* mutation positive advanced disease compared with platinum-doublet based chemotherapy regimens, from a U.S. health plan perspective (43). Increasing *EGFR* testing rates from 50 to 100% increased overall health plan expenditures by $0.013 per member per month (PMPM). Treatment costs contributed $0.012 PMPM with extended duration of treatment giving the greatest contribution. The cost of *EGFR* mutation testing was estimated at $0.002 PMPM, but was offset by the cost-savings associated with treatment of chemotherapy-related adverse events (−$0.002 PMPM).

Recent clinical data suggest an improvement in progression-free and overall survival for patients with *EGFR-*mutant NSCLC when treated with afatinib compared with platinum-based chemotherapy (44, 45). Although unable to estimate a plausible ICER based on the manufacturer’s submission, afatinib was considered to be a reasonable option for first or second-line treatment for patients with *EGFR*-mutant NSCLC, with exploratory estimates from the Evidence Review Committee of an ICER of £39,300/QALY gained with afatinib compared to pemetrexed/cisplatin in the overall population, and an ICER of £23,700/QALY gained in the non-Asian population based on trial data provided (46).

### ALK inhibitors

The cost-effectiveness of testing methodology for *EML4-ALK* fusion-positive tumors has been assessed using differing techniques, from a societal perspective using the US healthcare system (26). By varying *ALK* testing methods and population tested, the CE of FISH testing for all patients was estimated at $106,707USD/QALY, compared with $57,165USD/QALY for IHC. In a clinically selected population of non-smokers with *EGFR*- and *KRAS*-wild type adenocarcinoma, the CE estimates were $4,756USD/QALY and $2,548USD/QALY for FISH and IHC, respectively. One cost-effectiveness analysis has explored the use of crizotinib for the first-line treatment of patients with *EML4-ALK* fusion-positive tumors from the Canadian public healthcare perspective (22). The comparator was a platinum-doublet chemotherapy regimen in patients with non-squamous NSCLC, and the model incorporated subsequent treatment with pemetrexed and erlotinib. A re-biopsy strategy was employed in case of inadequate tissue. The method of assessment for EML4-ALK positive tumor was by initial IHC and, if positive, confirmatory testing with FISH. The incremental cost of crizotinib therapy for a gain of 0.11 QALYs was $2,725CAD/patient, with an ICER of $255,970/QALY gained. For patients with confirmed EML4-ALK positive tumors, first-line therapy with crizotinib produced an ICER of $250,632CAD/QALY, in excess of commonly accepted cost-effectiveness thresholds. Sensitivity analysis highlighted the major driver of cost as the price of crizotinib therapy. Despite FISH testing costs exceeding those of IHC, the relative cost of crizotinib was so great that use of the cost of initial FISH testing instead of IHC had minimal impact on the overall ICER.

### Histopathology

Patients with non-squamous NSCLC derive benefit from pemetrexed-based chemotherapy and from the addition of bevacizumab to a platinum-doublet. Although histologic subtype is not a recognized biomarker, these data have led to treatment selection for patients based on histologic subtype. Given the significant cost of these agents when compared with other standard chemotherapy regimens, several economic assessments have been performed. A cost–utility analysis of the addition of bevacizumab to platinum-based chemotherapy compared with chemotherapy alone (9) in patients with non-squamous NSCLC estimated an increase of 0.13 QALYs with the addition of bevacizumab, at a cost of $72,000USD per patient. The incremental cost–utility ratio for the addition of bevacizumab was $560,000USD/QALY (47), exceeding accepted thresholds for cost-effectiveness.

In the first-line setting, pemetrexed/cisplatin improved median overall survival by 1 month in advanced non-squamous lung carcinoma patients, when compared with gemcitabine/cisplatin, and an ICER estimated at £17,000–25,000/QALY (48). When pemetrexed is used as maintenance, the median survival gain compared to observation is 5 months, with an ICER of $122, 371USD/LYG.

## Conclusion

The management of lung cancer has transformed in recent years, due to increasing stratification of treatment based on pathologic and molecular characteristics. Optimizing treatment by using personalized therapy has resulted in improved treatment responses, quality of life, and progression-free survival of patients with NSCLC, with some evidence of survival benefit. However, this comes at a price, and, acknowledging that these actionable mutations are present only in a small subset of NSCLC tumors, we must act in the best interests of all our patients to ensure that this is affordable for the benefit gained.

In order to focus on the relevant population for a molecularly targeted therapy, tissue must be available and the testing method must be accurate. However, as in the case of *EML4-ALK*, there may be methods to select the target population with lower cost, and these technologies will continue to evolve. Further evolution of next-generation sequencing and multiplex platforms may also improve the cost-effectiveness of testing, where multiple abnormalities can be evaluated with a single test. While molecular testing beyond *EGFR* and *ALK* is not currently recommended as standard of care in NSCLC (18), more comprehensive genomic testing will likely become cheaper and more accessible in the future, minimizing time and tissue requirements in efforts to better personalize therapy (49, 50).

Cost-effective, -accurate, and -efficient methods of diagnosis must be employed, which allow equal accessibility to therapy for all patients. However, the major cost determinant in most economic evaluations of targeted treatment in NSCLC is drug price. Economic evaluations are integral to assessment of value for a given therapy as these may be used to enable funding decisions by policy-makers, and to negotiate pricing strategies with manufacturers where possible. There has been a paradigm-shift in the treatment of NSCLC with exciting new therapies revolutionizing treatment for patients with a previously dismal prognosis. As clinicians, we must ensure that as many patients as possible derive benefit from a personalized approach. Collaboration with payers and manufacturers is a key to ensure that cost of treatment is not prohibitive for patients and permitting further advances in lung cancer therapy.

## Conflict of Interest Statement

The authors declare that the research was conducted in the absence of any commercial or financial relationships that could be construed as a potential conflict of interest.
